# Obesity Is Less Frequently Associated with Cognitive Impairment in Elderly Individuals: A Cross-Sectional Study in Yogyakarta, Indonesia

**DOI:** 10.3390/nu12020367

**Published:** 2020-01-30

**Authors:** Amelia Nur Vidyanti, Muhammad Hardhantyo, Bayu Satria Wiratama, Astuti Prodjohardjono, Chaur-Jong Hu

**Affiliations:** 1 College of Medicine, Taipei Medical University, Taipei 11031, Taiwan; amelia.nur.v@ugm.ac.id; 2 Department of Neurology, Faculty of Medicine, Public Health, and Nursing, Universitas Gadjah Mada, Yogyakarta 55281, Indonesia; dr_astutisps@yahoo.co.id; 3 PhD Program of Public Health, College of Public Health, Taipei Medical University, Taipei 11031, Taiwan; hardhantyo@gmail.com; 4 Faculty of Health Science, Universitas Respati Yogyakarta, Yogyakarta 55281, Indonesia; 5 Graduate Institute of Injury Prevention and Control, College of Public Health, Taipei Medical University, Taipei 11031, Taiwan; bayu.satria@ugm.ac.id; 6 Biostatistics, Epidemiology and Population Health, Faculty of Medicine, Public Health, and Nursing, Universitas Gadjah Mada, Yogyakarta 55281, Indonesia; 7 Department of Neurology, School of Medicine, College of Medicine, Taipei Medical University, Taipei 11031, Taiwan; 8 Department of Neurology and Dementia Center, Shuang Ho Hospital, Taipei Medical University, New Taipei City 23561, Taiwan; 9 The PhD Program for Neural Regenerative Medicine, College of Medical Science and Technology, Taipei Medical University, Taipei 11031, Taiwan

**Keywords:** obesity, elderly, body mass index, cognitive impairment, Indonesia

## Abstract

Obesity is one of the factors associated with cognitive impairment. However, obesity may differently affect cognitive function in different age groups, and scarce data are available from low- and middle-income countries. This cross-sectional study aimed to identify the association between obesity and cognitive impairment among 143 elderly individuals in Yogyakarta. We recorded the sociodemographic factors and some comorbidities, also measured the body mass index as a parameter of obesity, cognitive function using Montreal Cognitive Assessment—Indonesia, mood condition and depression status using geriatric depression scale-short form, as well as the daily life function using Activity of Daily Living and Instrumental Activity of Daily Living. After adjustment for the sociodemographic and comorbidities, we found that subjects with older age were more likely to have cognitive impairment (odds ratio [OR] 3.544, 95%CI: 1.36–9.22, *p* < 0.01) and compared with elderly individuals with normal weight, obese elderly individuals were 40% less likely to have cognitive impairment (OR 0.604, 95%CI: 0.39–0.95, *p* < 0.05). This study suggests that obesity in elderly individuals is less frequently associated with cognitive impairment. These findings support the reverse causation mechanism related to body mass index (BMI) and cognitive impairment in low/middle-income countries.

## 1. Introduction

Normal aging, mild cognitive impairment (MCI), and severe cognitive impairment (dementia) serve as a continuous spectrum of cognitive function in elderly individuals [1]. Elderly individuals with cognitive impairment experience problems in recalling and remembering, focusing attention, learning new things, decision making skills, planning, judging, and language skills [2,3]. The difference between mild and severe cognitive impairments is the extent to which one’s daily living is affected by the impairment [3,4].

Some factors, such as education; socioeconomic status; obesity; and comorbidities including dyslipidemia, hypertension, diabetes mellitus, stroke, and cardiovascular disease, can affect cognitive function in elderly individuals [5,6]. Obesity has been established as a major public health concern worldwide, as it contributes to increased mortality and morbidity from cardiovascular diseases (CVD) [7]. Although obesity may also affect cognitive function, the underlying mechanism by which it leads to cognitive decline remains elusive. Evidence has shown that obesity affects cognitive function in different ways for different people based upon their age. Higher body mass index (BMI) in elderly individuals was associated with a decreased risk of dementia [8,9]. However, these findings were reversed in middle age, where higher BMI was associated with an increased risk of dementia [10,11,12]. These unexpected findings might possibly be caused by the reverse causation mechanism. Weight loss occurred steadily or progressively in elderly might be a sign of developing dementia in the near future [12]. Weight loss could begin earlier up to 20 years prior to symptoms onset and has been associated with preclinical and prodromal dementia stages, especially Alzheimer’s dementia [13,14].

The prevalence of obesity in low- and middle-income countries has rapidly increased, leading to an increased risk of CVD and its consequences [15]. However, the association between obesity and cognitive function in later life or among elderly individuals in these countries has not been well documented. A study from the 2007 Indonesian Family Life Survey (IFLS)-4 reported that obesity had a positive association with better cognitive function in adults aged 50 years and above [16]. Moreover, another study conducted in Yogyakarta, Indonesia reported that individuals aged above 65 years with increased level of cognitive impairment were likely to be underweight or malnourished [17]. Although this study did not explore the association between obesity and cognitive function, it evidenced the association between BMI and cognitive impairment.

The latest systematic review and meta-analysis of longitudinal studies reported that obesity in later life was negatively associated with dementia, and no heterogeneity between each study was observed. However, this review only included studies from high-income countries (mostly from the US, Europe, and Japan) [18], which might be attributed to the lack of well-documented longitudinal studies from low/middle-income countries demonstrating the association between obesity and cognitive impairment.

Indonesia is a lower middle-income country with 34 provinces. One of the provinces, Yogyakarta, has the greatest life expectancy and highest number of elderly individuals. This condition leads Yogyakarta to be a suitable province to study the aging population in Indonesia [19]. The present study identified the association between obesity and cognitive impairment. The results of this study may provide additional evidence of the association between obesity and cognitive impairment in older age, particularly in Indonesia.

## 2. Materials and Methods 

### 2.1. Study Design and Study Population

This study had a cross-sectional design and included community-dwelling elderly individuals residing in Sleman Regency of Yogyakarta, Indonesia. Among the five regencies in Yogyakarta, Sleman is the only regency that has both lowland and highland areas. Thus, residents in Sleman Regency represent the characteristics of both urban and rural population. Sleman Regency also has the highest population among other regencies in Yogyakarta [20]. Individuals >60 years old and residing in the Sleman Regency of Yogyakarta, Indonesia were eligible to participate in this study. Prior to data collection, the research team explained the study and provided a volunteer information sheet to the participants. All participants signed a written informed consent form prior to the investigation. For participants who were unable to understand the study and implications for participating in this study, the informed consent was provided by their spouse, child, or close relative. Individuals with aphasia, schizophrenia, visual impairment, or hearing problems and illiterate individuals were excluded.

We used cluster sampling to determine the study population. Three clusters (three out of 17 districts in Sleman Regency of Yogyakarta) were randomly selected using a computer-generated random number (GraphPad QuickCalcs, GraphPad Software Inc., La Jolla, CA, USA). Then, nine suburbs/villages within the three districts were selected as the final locations. Finally, individuals aged more than 60 years were randomly selected per suburb/village.

We estimated the minimum sample size and found that it might be acceptable to represent the entire population. The sample size was calculated using the following formula for cross-sectional studies [21]:

$$\frac{N\;={\;Z\alpha }^{2}\;\times \;P\;\times \;Q}{d^{2}}$$

*N* = sample size
*Zα* = the statistic corresponding to the level of confidence
*P* = expected prevalence (that can be obtained from the same studies or a pilot study conducted by the researchers)
*Q* = 1 − *P*
*d* = precision (corresponding to effect size)

No previous study has reported the prevalence of cognitive impairment in Indonesia. Therefore, we determined the expected prevalence (P) of cognitive impairment to be 15.2% based on a previous study in Singapore [22], which was in accordance with the data from other Asian populations [23,24]. With *Zα* = 1.96 and *d* = 0.06, the minimum sample size is as follows:

$$\frac{N\;=\;Z\alpha^{2}\;\times \;P\;\times \;Q}{d^{2}}$$

$$\frac{N\;={\left(1.96\right)}^{2}\;\times \;0.152\;\times \;0.848}{0.06^{2}}$$

N=137.55 ~ 138 participant

A total of 150 elderly individuals were investigated in this study. However, seven of them were excluded because they refused to be further examined during the interview. In total, 143 participants completed the examinations, generating a response rate of 95.33%.

Ethical approval for this study was obtained from the Medical and Health Research Ethics Committee of the Faculty of Medicine, Universitas Gadjah Mada, Indonesia (EC No: KE/FK/479/EC/2016).

### 2.2. Data Collection and Measurements

We conducted a cross-sectional survey through face-to-face interviews based upon a questionnaire. The questionnaire consisting of five components, namely sociodemographic and comorbidities measurement, geriatric depression scale-short form (GDS-SF) to measure depression level in elderly individuals [25], Montreal Cognitive Assessment-Indonesia version (MoCA-INA) to assess the cognitive function [26], Activity of Daily Living (ADL) for measuring daily activities [27], and Instrumental Activity of Daily Living (IADL) for measuring dependency on daily living instruments [28]. All the questionnaires were filled in by the investigators. Approximately one hour was required by each participant to finish the interview and examination. The process of data collection was done during 09.00–12.00 in the morning and the participants should already had breakfast, were not asleep and free from any pain.

BMI was measured by calculating the body weight (in kilograms) divided by the square of height (in meter squares). The measurements were performed using a portable scale and stature meter. The classification of BMI in this study was based on WHO criteria of BMI for the Asian population: those with BMI < 18.5 kg/m^2^ were categorized as underweight, 18.5–22.9 kg/m^2^ as normal, 23–27.4 kg/m^2^ as overweight, and ≥27.5 kg/m^2^ as obese [29] (Supplementary Table S1).

Sociodemographic measures included age (60–65 years and more than 65 years which was symbolized with 65+), sex (female and male), socioeconomic status (SES) (low, middle, and high), number of children (≤3 and >3), and history of smoking.

Socioeconomic status (SES) was defined by measuring the three most commonly used indicators for SES: education, occupation, and income [30]. We made a composite index based on the three indicators, as in a previous study [31]. We adjusted the income variable based on the classification from Statistics Indonesia [32]. In the previous study, each indicator was scored from one to five. However, as the income variable used in the present study had only four levels and as we excluded uneducated or illiterate individuals, we made some modifications by adjusting the total cut-off score. The total score ranged from three to 12; a score of three to five indicated low SES, six to nine indicated middle SES, and 10 to 12 indicated high SES (Supplementary Table S1).

Comorbidities included a self-reported history of hypertension, hypercholesterolemia, cardiovascular disease, and traumatic brain injury (TBI). The blood pressure of each participant was also measured to verify the hypertension status.

To determine the cognitive impairment status of the participants, we examined the cognitive function and functional status by using four assessment tools, namely MoCA-INA, GDS-SF, ADL, and IADL. MoCA detects mild cognitive impairment with high sensitivity and specificity [33]. The Indonesia version of MoCA (MoCA-INA) has been validated as a cognitive screening tool in Indonesia and has been widely used in the clinical and research setting [26]. GDS-SF has been validated as a screening tool to measure the level of depression in elderly individuals [25]. ADL and IADL are the screening tools to assess functional status in patients with cognitive impairment by measuring their daily activities and dependency on the daily living instrument [27,28]. A combination of the aforementioned four assessment tools categorized whether participants had a normal cognitive function (without cognitive impairment), mild cognitive impairment (MCI), or severe cognitive impairment (dementia) [4,34,35] (Supplementary Table S1).

### 2.3. Statistical Analysis

Participants with MCI and severe cognitive impairment (dementia) were categorized together as having cognitive impairment (MoCA-INA ≤ 25). The Chi square test was used to analyze the statistical difference between categorical variables. The crude odds ratio of the factors associated with cognitive impairment was measured using univariate logistic regression analysis. These factors included BMI, sociodemographic status (age, sex, number of children, nutrition status, and smoking) and comorbidities (hypertension, hypercholesterolemia, cardiovascular disease, TBI). Finally, multiple logistic regression analysis was performed to measure the contribution of BMI to cognitive impairment after controlling covariates such as sociodemographic status and comorbidities for Models Two and Three, respectively. Interaction analysis of age and BMI was also performed to observe the influence of age on the main effect of BMI and cognitive impairment. A *p* value of <0.05 indicated statistical significance. SAS version 9.4 was used for all the analyses.

## 3. Results

### 3.1. BMI, Sociodemographic Characteristics, and Comorbidities

Table 1 presents the baseline characteristics of the study participants which consist of BMI, sociodemographic characteristics, and comorbidities. In total, of the 143 elderly individuals examined, 108 had cognitive impairment and 35 did not have cognitive impairment. There were significant associations between age, BMI, and SES with cognitive impairment status. However, no significant association between comorbidities and cognitive impairment status was observed.

### 3.2. Factors Associated with Cognitive Impairment

Table 2 presents the univariate analysis of factors associated with cognitive impairment. No significant differences in some sociodemographic characteristics and comorbidities were observed. Sex, number of children, smoking, hypertension, hypercholesterolemia, cardiovascular disease, and TBI had no association with cognitive impairment. Obese elderly individuals were approximately 58% less likely to have cognitive impairment compared with their normal-weight counterparts (odds ratio [OR] 0.424, 95%CI: 0.23–0.77, *p* < 0.01). Elderly individuals aged above 65 years (65+) were more likely to have cognitive impairment than those aged 60–65 years were (OR 3.513, 95%CI: 1.52–8.07, *p* < 0.01). Individuals with high SES were approximately 85% less likely to have cognitive impairment compared with their counterparts with low SES (OR 0.151, 95%CI: 0.04–0.55, *p* < 0.05).

Table 3 presents the multiple logistic linear analyses of factors associated with cognitive impairment after adjustment by controlling multiple covariates. In Model Two, we controlled the sociodemographic characteristics only and showed that the association of cognitive impairment in obese elderly individuals increased from 0.424 to 0.570. For Model Three, we adjusted the odds ratio of obesity by controlling the sociodemographic characteristics and comorbidities and found that individuals with obesity alone were about 40% less likely to have cognitive impairment (OR 0.604, 95%CI: 0.39–0.95, *p* < 0.05). Elderly individuals aged 65+ years were more likely to have cognitive impairment than those aged 60–65 years were (OR 3.544, 95%CI: 1.36–9.22, *p* < 0.01). Although SES was not a significant factor associated with cognitive impairment, it showed a strong reduction in cognitive impairment as SES increased (OR 0.128, 95%CI: 0.02–0.59, *p* 0.092). Interaction analysis of age and BMI with cognitive impairment revealed a significant interaction effect between obesity and age 60–65 years. Obese participants aged 60–65 years were 95.7% less likely to have cognitive impairment (OR 0.043, 95%CI: 0.003–0.536, *p* < 0.05) compared with participants with normal weight who were aged 60–65 years.

## 4. Discussion

In the present study, we demonstrated that cognitive impairment among elderly individuals in Yogyakarta, Indonesia was affected by age, SES, and BMI. Elderly aged 65+ years were positively associated with cognitive impairment, with high SES could be a strong factor to predict better cognitive function. Furthermore, to our knowledge, this study is the first study in Indonesia which showed that obesity in late life was less likely associated with cognitive impairment.

The present study showed that elderly individuals aged 65+ were more likely to have cognitive impairment. This finding is consistent with previous studies [36,37,38]. After performing the interaction analysis between age and BMI with cognitive impairment, we found that only obese elderly individuals aged less than 65 years (or those in the age range of 60–65 years) were significantly less likely to have cognitive impairment. This could be attributed to the fact that subjects in the present study had an average age of 69.38 years, which meant that they were relatively younger than the subjects in prior studies (average age >70 years) [36,37,38].

In addition to that, the illiteracy rate among elderly in Indonesia is still high. The illiteracy rate for elderly aged 65+ in the study area (Yogyakarta) in 2017 was 23.55% compared with 21.81% of those with the same age in whole Indonesia [39,40]. Therefore, there is still probability for very old people with cognitive impairment in the study area who were excluded due to illiteracy. This could also contribute to lack of significant association for being obese and 65+ and having cognitive impairment.

A substantial proportion of the participants (108 out of 143 or 75.5%) were classified as having cognitive impairment. The finding is in accordance with previous study conducted at urban and rural population in Yogyakarta, Indonesia by Arjuna et al. (2017) [17]. They found that 59% (in urban) and 80% (in rural) of their elderly subjects were cognitively impaired. In the present study, we recruited the participants residing in Sleman Regency, a region that represents the characteristics of both urban and rural population in Yogyakarta. Hence, the finding that 75.5% of our participants were cognitively impaired corroborate previous findings [17]. The higher case of cognitive impairment in older individuals aged ≥65 years in our study might due to most of them (51 out of 108) had low SES (low educational background, low income, and low level of occupation). However, this still needs further investigation to delineate the causal relationship.

Socioeconomic status (SES) has been known to be an important factor associated with cognitive impairment. Studies have shown that individuals with high SES were less likely to have cognitive impairment [41,42,43]. In the present study, on analysis of Model Two, we found that elderly with high SES were less likely to have cognitive impairment. However, in the final analysis, as shown in Model Three, we did not find any significant association between SES and cognitive impairment although SES showed as a strong predictor for cognitive function in elderly. Nevertheless, this finding warrants further investigation.

In the present study, we found that obesity in elderly individuals was less likely associated with cognitive impairment. This finding corroborates those of previous studies, as elderly individuals with low BMI (<25 kg/m^2^) and those who were losing weight had a higher risk of dementia and showed more rapid cognitive decline [12,44,45]. Furthermore, the other studies have shown that obesity was associated with a lower risk of dementia among older individuals [9,46,47]. Obesity or higher BMI in the elderly individuals may come from the higher muscle mass or increased fat accumulation in regions other than the abdominal area; for instance, leg fat mass [47]. Greater leg fat mass in elderly individuals has been associated with improved glucose metabolism [48], which eventually reduces the risk of cognitive impairment [49].

Prior studies have reported that obesity in later life was associated with a lower risk of cognitive impairment, whereas obesity in midlife was associated with a higher risk of cognitive impairment. This contradictory finding is called obesity paradox caused by reverse causation which may be explained by several mechanisms proposed by researchers [12]. First, low body weight may indicate weight loss caused by underlying pathology related to general health deterioration and the progression of dementia [50,51]. The preclinical phase of dementia, which involves very mild cognitive impairment, could last several years. Weight loss, one of the signs of Alzheimer’s dementia, could begin much earlier before the clinical symptoms of dementia become apparent [52,53]. Previous studies have found that patients with Alzheimer’s dementia had reduced olfaction, dysfunction in the limbic system and hypothalamus, and reduced automaticity in swallowing and chewing [12,54]. This could lead to loss of appetite and weight loss. Weight loss may also be related to the neuropsychiatric symptoms that are commonly observed in the early phase of dementia, such as loss of initiative and apathy [55]. Second, weight loss is in conjunction with assorted criteria of frailty in which frailty is associated with diminished performance of cognitive function [56]. Another explanation, excess body weight provides more energy savings and a stronger inflammatory response that could be a benefit to encounter acute illness [57]. Some evidences from neuroimaging studies found that higher BMI in dementia patients with Alzheimer’s disease (AD) was associated with greater volumes of medial temporal cortex which indicate better cognitive performance [58]. Another study revealed higher BMI in older individuals with AD was associated with higher glucose metabolism in the anterior cingulate gyrus and hypothalamus which was related to better cognitive function [59]. An additional explanation suggests that higher BMI in late-life individuals was associated with functional brain connectivity which served as a neuroprotection for cognition [60]. Nevertheless, the exact mechanism by which obesity was less frequently associated with cognitive impairment in elderly individuals in the present study remains unknown.

We also found that none of the comorbidities had any association with cognitive impairment. This finding is contradictory with those of the previous studies, which showed that hypertension, diabetes mellitus, stroke, and CVD were associated with a higher risk of cognitive impairment [61,62,63]. This discrepancy might be due to most of the history of comorbidities in the present study were self-reported. Although we also measured the blood pressure to verify the history of hypertension, the other comorbidities were only verified by the subject use of medications if they still consumed it. This self-reported information could lead to exposure bias.

The present study contributes to providing further evidence for a negative association between obesity and cognitive impairment in older individuals from low/middle-income countries. This finding may facilitate a more comprehensive longitudinal study regarding the link between obesity and cognitive impairment in late life, particularly in Indonesia. Nevertheless, this study has several limitations. First, it only included participants from one specific area in Yogyakarta and had a small sample size owing to limited funding. Hence, the findings in the present study require careful interpretation upon generalization. Second, only BMI was measured as a single parameter for obesity. Using BMI as a single marker of obesity is considered a poor indicator of cardiometabolic health. Obesity in the elderly should be adjusted for body composition [64]. Body composition, which consists of body mass (skeletal muscle mass), fat mass, and fat-free mass, has been suggested to affect the central nervous system that controls cognition, motivation, and executive function [65]. The reason why we only measured BMI in the present study is because we refer to Guideline of Nutritional and Health Service for Elder People by Ministry of Health of Indonesia (Pedoman Pelayanan Gizi Lanjut Usia, Kementerian Kesehatan Republik Indonesia, 2012) [66]. Based on the guideline, it is stated that BMI could be used as a simple initial screening tool for measuring nutritional status of elderly in Indonesia. For further detailed examination of nutritional status, BMI combined with Mini Nutritional Assessment (MNA) followed by the measurement of waist circumference, waist-to-height ratio, waist/hip ratio, skinfold thickness, or body fat percentage could be achieved [66]. Although only using BMI as a single indicator, we tried to optimize the interpretation by adjusting BMI cut-off point based on characteristics of Indonesian population. The BMI cut-off point of obesity for Indonesian population is lower than the common cut-off point used in Western countries, which is ≥27.5. This finding comes from the different associations between BMI, percentage of body fat, and health risk than do Western populations [29]. Indonesian populations having the same weight, height, age, and sex generally have higher percentage of body fat compared to Western populations [67]. Third, the information about history of comorbidities were only self-reported without any verification from medical records. This could lead to exposure bias and hinder the association between comorbidities and cognitive impairment. Finally, the present study only used cross-sectional data, which could not establish the causal relationship between obesity and cognitive impairment, although the direction of the influence was consistent with previous research. More knowledge on this issue is needed in the Asian countries, to better plan preventive interventions for cognitive impairment in elderly population.

## 5. Conclusions

Cognitive impairment was positively associated with older age and negatively associated with higher BMI among elderly individuals in Indonesia. Elderly with late life obesity was less likely to have cognitive impairment which supports the obesity paradox. However, the mechanism underlying this phenomenon warrants further investigations.

## Figures and Tables

**Table 1:** Body mass index (BMI), sociodemographic characteristics, and comorbidities of the participants.

Characteristics	No Cognitive Impairment		Cognitive Impairment		*p* Value
	N	%	N	%	
Body mass index					<0.05 *
Underweight	1	2.86	17	15.74	
Normal	15	42.86	48	44.44	
Overweight	5	14.29	24	22.22	
Obese	14	40	19	17.59	
Age (years)					<0.01 **
60–65	15	42.86	19	17.59	
65+	20	57.14	89	82.41	
Sex					0.277
Female	16	45.71	62	57.41	
Male	19	54.29	46	42.59	
Socioeconomic status					<0.01 **
Low	6	17.14	51	47.22	
Middle	22	62.86	48	44.44	
High	7	20	9	8.33	
Children					0.517
≤3	9	25.71	34	31.48	
>3	26	74.29	74	68.52	
Smoking					0.398
No	26	74.29	72	66.67	
Yes	9	25.71	36	33.33	
Hypertension					0.861
No	21	60	63	58.33	
Yes	14	40	45	41.67	
Hypercholesterolemia					0.059
No	24	68.57	90	83.33	
Yes	11	31.43	18	16.67	
Cardiovascular disease					0.964
No	32	91.43	99	91.67	
Yes	3	8.57	9	8.33	
Traumatic brain injury					0.971
No	33	94.29	102	94.44	
Yes	2	5.71	6	5.56	

* *p* < 0.05; ** *p* < 0.01.

**Table 2:** Univariate analysis of factors associated with cognitive impairment.

Characteristics	Odds Ratio	95%CI	*p* Value
Body mass index			
Underweight	5.312	(0.65–43.31)	0.083
Normal	Ref		
Overweight	1.500	(0.48–4.61)	0.827
Obese	0.424	(0.23–0.77)	<0.01 **
Age (y.o)			
60–65	Ref		
65+	3.513	(1.52–8.07)	<0.01 **
Sex			
Female	Ref		
Male	0.625	(0.29–1.34)	0.229
Socioeconomic status			
Low	Ref		
Middle	0.257	(0.09–0.68)	0.322
High	0.151	(0.04–0.55)	<0.05 *
Children			
≤3	Ref		
>3	0.753	(0.31–1.78)	0.518
Smoking			
No	Ref		
Yes	1.444	(0.61–3.40)	0.400
Hypertension			
No	Ref		
Yes	1.071	(0.49–2.33)	0.861
Hypercholesterolemia			
No	Ref		
Yes	0.436	(0.18–1.04)	0.063
Cardiovascular disease			
No	Ref		
Yes	0.970	(0.24–3.80)	0.964
Traumatic brain injury			
No	Ref		
Yes	0.970	(0.18–5.04)	0.971

* *p* < 0.05; ** *p* < 0.01.

**Table 3:** Multiple logistic linear analyses of factors associated with cognitive impairment after adjustment for the covariates.

Variable (Risk vs. Reference)	OR	95%CI	*p* Value
Model One			
Body mass index (obese vs. normal)	0.424	(0.23–0.77)	<0.01 **
(underweight vs. normal)	5.321	(0.65–43.31)	0.083
(overweight vs. normal)	1.500	(0.48–4.61)	0.827
Model Two			
Body mass index (obese vs. normal)	0.570	(0.37–0.88)	<0.01 **
(underweight vs. normal)	6.982	(0.76–63.50)	0.090
(overweight vs. normal)	2.328	(0.64–8.37)	0.564
Age (65+ vs. 60–65 years)	3.459	(1.35–8.85)	<0.01 **
Sex (male vs. female)	0.389	(0.12–1.18)	0.096
Socioeconomic status (high vs. low)	0.108	(0.02–0.49)	<0.05 *
Children (>3 vs. ≤3)	0.786	(0.29–2.13)	0.636
Smoking (smoker vs. nonsmoker)	0.640	(0.19–2.09)	0.461
Model Three			
Body mass index (obese vs. normal)	0.604	(0.39–0.95)	<0.05 *
(underweight vs. normal)	7.577	(0.78–72.86)	0.096
(overweight vs. normal)	2.289	(0.61–8.48)	0.638
Age (65+ vs. 60–65)	3.544	(1.36–9.22)	<0.01 **
Sex (male vs. female)	0.386	(0.12–1.20)	0.100
Socioeconomic status (high vs. low)	0.128	(0.02–0.59)	0.092
Children (>3 vs. ≤3)	0.618	(0.21 – 1.82)	0.382
Smoking (smoker vs. nonsmoker)	0.631	(0.19–2.09)	0.451
Hypertension (yes vs. no)	1.091	(0.42–2.83)	0.858
Hypercholesterolemia (yes vs. no)	0.490	(0.15–1.52)	0.218
Cardiovascular disease (yes vs. no)	0.675	(0.14–3.21)	0.621
Traumatic brain injury (yes vs. no)	0.987	(0.11–8.35)	0.990
Interaction analysis: age and BMI with cognitive impairment ^a^			
Normal and age 60–65 years	1		
Normal and age 65+ years	1.238	0.270–5.680	0.783
Underweight and age 60–65 years	(empty)		
Underweight and age 65+ years	7.001	0.561–87.389	0.131
Overweight and age 60–65 years	1.462	0.142–15.103	0.750
Overweight and age 65+ years	3.068	0.458–20.577	0.248
Obese and age 60–65 years	0.043	0.003–0.536	0.015
Obese and age 65+ years	1.968	0.312–12.418	0.471

* *p* < 0.05; ** *p* < 0.01. ^a^ Adjusted for sex, socioeconomic status, number of children, smoking status, hypertension, hypercholesterolemia, cardiovascular disease, and traumatic brain injury.

## Supplementary Materials

The following are available online at https://www.mdpi.com/2072-6643/12/2/367/s1, Table S1: SES, cognitive impairment, and BMI assignments.

## Abbreviations

MCI	mild cognitive impairment
CVD	cerebrovascular disease
IFLS	Indonesia Family Life Survey
GDS-SF	Geriatric Depression Scale-Short Form
MoCA-INA	Montreal Cognitive Assessment-Indonesia version
ADL	Activity of Daily Living
IADL	Instrumental Activity of Daily Living
BMI	body mass index
SES	socioeconomic status
TBI	traumatic brain injury
AD	Alzheimer’s disease
