# Supplementary material for: Obesity Is Less Frequently Associated with Cognitive Impairment in Elderly Individuals: A Cross-Sectional Study in Yogyakarta, Indonesia
Source: Nutrients. 2020 Jan 30;12(2):367. doi: 10.3390/nu12020367 (PMC7071195; doi:10.3390/nu12020367)
Supplement: Supplementary file 1 [file nutrients-12-00367-s001.pdf]

Table S1. SES, cognitive impairment, and BMI assignments.

Classification

1. Body mass index (BMI) (kg/m^2)

Score			Categories
<18.5	-	-	Underweight
18.5–22.9	-	-	Normal
23–27.4	-	-	Overweight
≥27.5	-	-	Obese

2. Socioeconomic status (SES)

Personal Monthly Income (IDR)	Educational level	Occupation	Score
<1.000.000	Elementary school	Laid-off workers	1
1.000.000–2.000.000	Junior high school	Retirees	2
2.000.000–3.000.000	Senior high school	Self-working/service industries	3
>3.000.000	University or Above	Enterprise/Civil Servants	4

3. Cognitive function

MoCA–INA‡	GDS-SF‡	ADL or IADL‡	Categories
>25	≤5	0 or 0	Without cognitive impairment
19–25	≤5e	0 or ≤2	Mild cognitive impairment

<19	-	≥1 or >2	Severe cognitive impairment (dementia)

[‡]MOCA–INA = Montreal Cognitive Assessment-Indonesia version; GDS-SF = Geriatric Depression Scale-Short Form; ADL = Activity of Daily Living; IADL = Instrumental Activity of Daily Living
